# Specific expression of novel long non-coding RNAs in high-hyperdiploid childhood acute lymphoblastic leukemia

**DOI:** 10.1371/journal.pone.0174124

**Published:** 2017-03-27

**Authors:** Mathieu Lajoie, Simon Drouin, Maxime Caron, Pascal St-Onge, Manon Ouimet, Romain Gioia, Marie-Hélène Lafond, Ramon Vidal, Chantal Richer, Karim Oualkacha, Arnaud Droit, Daniel Sinnett

**Affiliations:** 1 Division of Hematology-Oncology, Research Center, Sainte-Justine University Health Center, 3175 Chemin de la Côte-Sainte-Catherine, Montréal, QC, Canada; 2 Mathematics and Statistics Department, University of Quebec at Montreal (UQAM), 201 President-Kennedy Av., Montreal, QC, Canada; 3 Department of Endocrinology and Nephrology, Laval University, 2705 Laurier Blvd., Quebec City, QC, Canada; 4 Department of Pediatrics, Faculty of Medicine, University of Montreal, Montreal, QC, Canada; Washington State University, UNITED STATES

## Abstract

Pre-B cell childhood acute lymphoblastic leukemia (pre-B cALL) is a heterogeneous disease involving many subtypes typically stratified using a combination of cytogenetic and molecular-based assays. These methods, although widely used, rely on the presence of known chromosomal translocations, which is a limiting factor. There is therefore a need for robust, sensitive, and specific molecular biomarkers unaffected by such limitations that would allow better risk stratification and consequently better clinical outcome. In this study we performed a transcriptome analysis of 56 pre-B cALL patients to identify expression signatures in different subtypes. In both protein-coding and long non-coding RNAs (lncRNA), we identified subtype-specific gene signatures distinguishing pre-B cALL subtypes, particularly in t(12;21) and hyperdiploid cases. The genes up-regulated in pre-B cALL subtypes were enriched in bivalent chromatin marks in their promoters. LncRNAs is a new and under-studied class of transcripts. The subtype-specific nature of lncRNAs suggests they may be suitable clinical biomarkers to guide risk stratification and targeted therapies in pre-B cALL patients.

## Introduction

Pre-B cell childhood acute lymphoblastic leukemia (pre-B cALL) is the most frequent pediatric cancer, representing ~25% of all cases. Prognosis is based on the absence or the presence of chromosomal rearrangements or gross aneuploidy [1,2]. High hyperdiploidy (HeH) cases, defined as having >50 chromosomes [3,4], and the t(12;21)[*ETV6/RUNX1*] rearrangement represent together nearly half of the chromosomal anomalies encountered in pre-B cALL and are associated with a favorable outcome [5,6]. Other subtypes, such as *MLL*-rearranged, t(1;19)[*TCF3/PBX1*], or t(9;22)[*BCR/ABL1*] are seen at much lower frequencies (<10%) and are associated with intermediate-to-poor outcomes [1,2]. Despite the availability of these molecular and chromosomal markers, accurate patient risk stratification is an ongoing challenge in cALL treatment. Indeed, karyotyping requires the observation of multiple cells undergoing mitosis, which are not always available or present in bone marrow or peripheral blood smears. Furthermore, current molecular approaches used in the detection of known chromosomal rearrangement yielding chimeric proteins, although highly sensitive, are not suitable for disease subtypes lacking these fusion products.

Several studies have described expression signatures for classification of molecularly-defined ALL subtypes and improved outcome prediction [7–14]. These studies focused on the analysis of protein-coding transcripts, probably because most of their translated proteins are important signaling molecules.

A new class of non-coding RNAs, designated as long ncRNAs (lncRNAs) have been recently described. LncRNAs play important regulatory roles in various biological processes including cell pluripotency and tumorigenesis [15–18]. LncRNAs can exert their effects through mechanisms such as chromatin remodeling, *cis* regulation at enhancers and post-transcriptional regulation of mRNA processing [18]. A recent microarray-based study has identified several lncRNAs differentially expressed in pre-B cALL that discriminate the t(12;21)[*ETV6*/*RUNX1*], t(1;19)[*TCF3*/*PBX1*], and *MLL*-rearranged tumor subtypes [19]. This study was limited to a number of known lncRNA loci and thus does not allow for the identification of novel transcripts or isoforms.

In this study, a full transcriptome analysis of a cohort of 56 pre-B cALL patients demonstrated that both specific protein-coding and lncRNA transcription signatures could accurately discriminate between pre-B cALL subtypes. In addition, we showed that epigenetic changes at the promoters of protein-coding and lncRNA genes deregulated in pre-B cALL are both enriched in bivalent histone marks. This study showed that lncRNA expression signatures might constitute useful molecular biomarkers for pre-B ALL subtypes stratification.

## Results

### Differential expression analysis identified subtype-specific transcripts in cALL

We analyzed the transcriptomes of 56 pre-B cALL patients (see **Table 1** for patients’ characteristics) to identify transcription-based molecular biomarkers. For comparison we used 3 control samples corresponding to CD10^+^CD19^+^ pre-B cells isolated from human cord blood (HCB).

**Table 1:** Cohort characteristics.

Subtype	ID	Sex	Blast Rate (%)	Age (months)	DNA Index	Karyotype	Prognostic Risk Group	Events (R = Relapse, D = Death)	DFCI Protocol
HHD	315	F	98	127	1.12		High	R	95–01
	327	M	98.5	72	1.13		Standard		95–01
	39	M	-	49	1.14		Standard		91–01
	442	M	95.5	48	1.12	4,6,21,X	High		2000–01
	659	M	99.5	35	1.16	2,4,6,10,14,18,21,X 3?	High	R	2000–01
	670	M	92.5	33	1.2	2,4,6,9,10,14,17,21,X	High	R	2000–01
	777	F	89	33	1.15	4,6,10,14,17,21	High		2005–01
	801	M	93	48	1.17	4,6,8,10,14,17,18,21,X	High		2005–01
	813	F	74	51	1.18	4,5,6,10,14,17,21,X	Standard		2005–01
	819	M	97	48	1.12	4,6,14,21	High		2005–01
	826	F	50.2	13	1.25	4,5,6,7,8,10,11,12,14,15,17,21,22	Standard		2005–01
Other	399	M	96.5	43	1	8,X	Standard	R,D	95–01
	41	M	-	127	1.07		High		91–01
	419	M	93.5	172	1	None	High		2000–01
	436	F	94.5	99	1	None	Standard		2000–01
	446	F	90.5	106	-	None	High		2000–01
	447	F	97.5	129	1	21c	High	R,D	2000–01
	553	M	99.4	81	1	None	Standard		2000–01
	579	M	95.5	75	1	None	High	R,D	2000–01
	580	F	97	52	1		Standard		2000–01
	595	M	97	155	1	5	High	R,D	2000–01
	596	F	96.5	22	1	None	Standard		2000–01
	599	M	100	88	1	None	High		2000–01
	608	M	83	47	1	None	High		2000–01
	617	F	93	46	1		Standard		2000–01
	720	F	94.5	37	1	None	Standard	R,D	2005–01
	756	F	92.5	30	1	None	High		2005–01
	757	F	96.5	127	1	None	High		2005–01
	794	M	100	168	1		High		2005–01
	831	F	91	187	1	X	High		2005–01
t(12;21)	220	M	92.5	69	1		High		95–01
	373	F	97.5	55	1		High		95–01
	392	M	97	84	1	None	Standard		95–01
	411	F	86.8	57	1	None	Standard		95–01
	413	F	83.2	40	1	None	Standard		2000–01
	420	M	97.5	57	1		High		2000–01
	443	F	99.5	32	1	None	High		2000–01
	5	M	-	63	1		Standard		91–01
	614	F	96	48	1	None	High		2000–01
	676	F	93	43	1		Standard		2000–01
	691	M	97.5	30	1		Standard		2005–01
	696	F	98	51	1		Very High		2005–01
	73	F	-	78	1		Standard		95–01
	732	F	96	33	1	None	Standard		2005–01
	753	F	95	61	1	None	Standard		2005–01
	814	F	93	107	1	None	High	R,D	2005–01
	817	F	93	67	1	None	Standard		2005–01
	824	M	87	66	1	None	Standard		2005–01
	827	M	76.5	45	1	21	High		2005–01
	832	M	98.5	61	1		Standard		2005–01
	854	F	91.5	31	1	None	Standard		2005–01
	856	M	92	30	1	None	Standard		2005–01
t(9;22)	485	M	96.4	77	1	None	High	R,D	2000–01
	697	M	96	113	1	None	High	D	2005–01
	790	M	88.5	171	1	Not enough information	High	R	-
	825	F	91	138	1	None	High		2005–01

**Fig 1:**
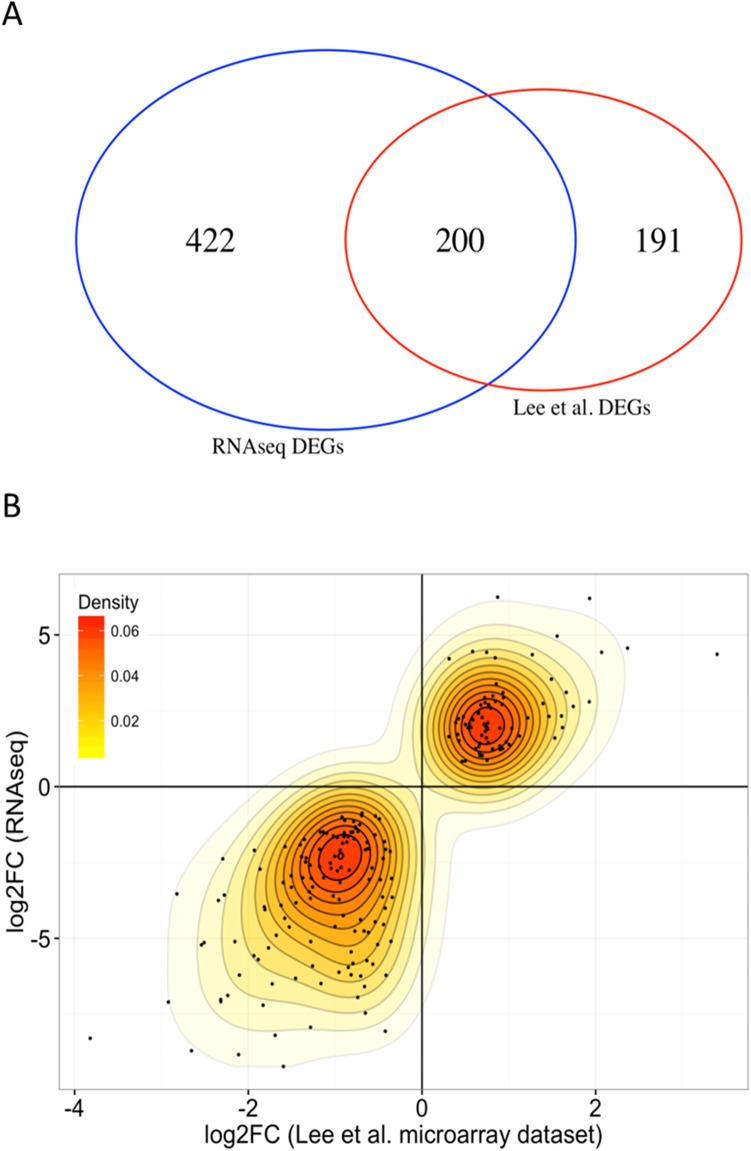
Comparison of differentially expressed genes in our RNA-seq and public dataset. (A) Overlap between differentially expressed genes identified from microarray data (Lee et al.) and RNA-seq for the HeH versus t(12;21) comparison. The intersection of 200 genes represents a 10-fold enrichment compared to the expected intersection (20) when DEGs are picked randomly. (B) Comparison of logFCs for DEGs identified in both the microarray and RNA-seq analysis. Pearson’s product-moment correlation between log2FCs = 0.844. Spearman’s rank correlation = 0.793. We note that expression changes are coherent (in the same direction) for all DEGs identified from both datasets

We identified a total of 4130 transcripts that were differentially expressed (DET) in at least one molecular subtype (t(12;21), t(9;22), HeH, or Others) relative to HCB controls (**S1 Table**). Of these, 1624 were either up- or down-regulated in all four subtype categories relative to HCB (“leukemia-specific” transcripts), whereas 438 were up- or down-regulated in only one subtype (“subtype-specific” transcripts). We then compared our DETs with publicly available microarray pre-B cALL expression data [20]. Since the controls used in that study were different from ours, we restricted our analysis to 472 protein-coding genes differentially expressed (FDR = 1e-3) between the t(12;21) and HeH subtypes in their dataset. Of these, 391 were expressed in our dataset, including 200 that were differentially expressed in both datasets (**S2 Table**), representing a 10-fold enrichment to the expected overlap size if DEGs were picked randomly (**Fig 1A**). Interestingly, we observed a strong correlation (Pearson's r = 0.84; p-value<2.2e-16) for these 200 genes both in terms of fold change and direction of change (up- or down-regulated) (**Fig 1B**). This indicates a strong transcriptional specificity of the t(12;21) and HeH subtypes since the results originate from different samples and were processed on different platforms.

**Fig 2:**
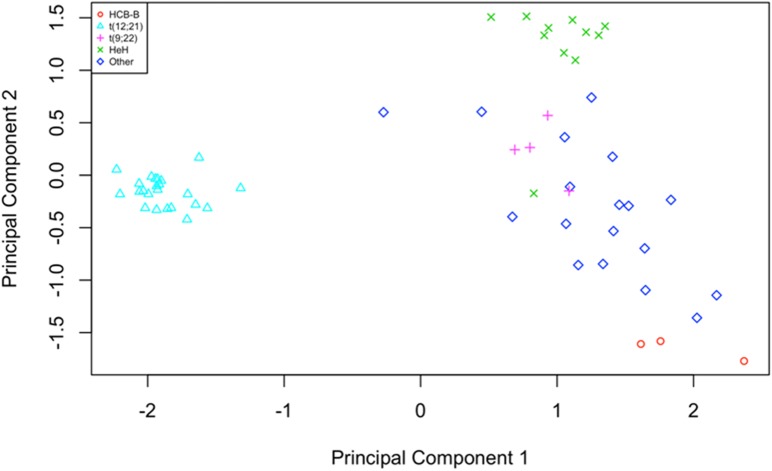
Multidimensional scaling plot of distances between gene expression profiles. The distance between each pair of samples is the Euclidean distance between expression values (logCPM) of the 500 genes with the most variance across all samples. Samples with an unknown phenotype or belonging to a cALL subtype appearing less than four times in our cohort have been labelled as “Other”.

Multidimensional scaling (MDS) analysis revealed distinct disease subtype-specific clusters (**Fig 2**). Similar subtype-specific expression patterns had also been reported in other studies [7,8,21,22]. In this study, most high hyperdiploid (HeH) patients (10/11) clustered together. In addition, we observed a well-defined cluster regrouping patients bearing the t(12;21) translocation. Of note, patients P5, P73, and P220, which clustered with t(12;21) samples, initially had unknown molecular subtype. Subsequent RT-PCR analysis reassigned them as t(12;21).All other rarer molecular subtypes, collectively labeled as “Other”, showed no clear clustering, probably due to the diversity of subtypes and limited number of each subgroup. Interestingly, although part of this diffuse “Other” group, the four t(9;22) patients were located close to each other, implying some similarity between their transcriptional profiles.

### Transcriptional signatures can classify pre-B cALL-specific subtypes

**Fig 3:**
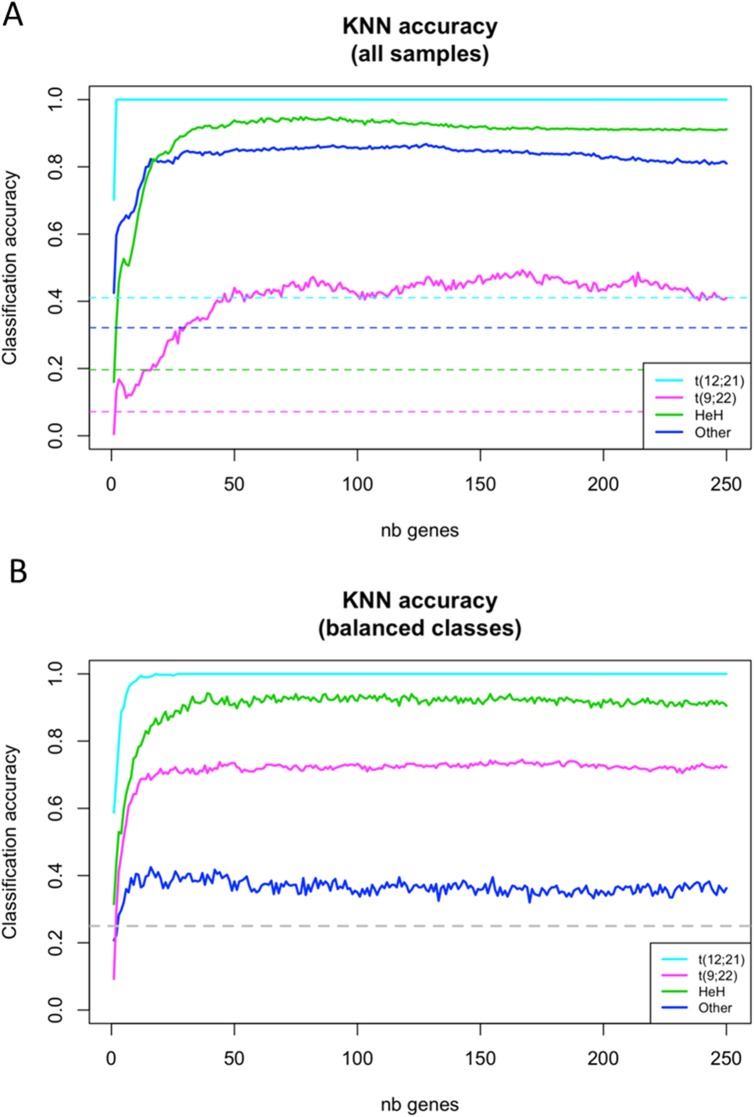
Accuracy of k-nearest neighbors (KNN) classification according to the number of considered top variances genes. Each continuous line gives the fraction of tumor samples correctly classified by cALL subtype, averaged over 100 replicates. For each replicate, we sampled 50% from all genes and ordered them according to expression (logCPM) variance across samples. KNN (3-nearest neighbors) classification was then performed, considering Euclidean distance between samples based on an incremental number of genes (pseudogenes excluded). (A) Leave-one-out classification was performed using all tumor samples. (B) Under-sampling was performed so that four tumor samples from each subtype were used at each iteration. Dashed lines show the expected accuracies when predictions are made by random assignation of cALL subtype

We next wanted to estimate the accuracy of expression-based cALL subtype prediction and determine how many genes are required for this task. To mitigate the risk of data over-fitting, we used the k-nearest neighbors (KNN) classification algorithm with leave-one-out cross validation [23](without considering tumor subtype *a priori*). We found that as few as 50 genes were needed for accurate subtype classification, particularly for the t(12;21) and HeH subtypes (**Fig 3A**).

While classification of t(12;21) patients quickly reaches 100% accuracy, the classification of t(9;22) patients only reaches 44% at 50 genes. This could be explained by class imbalance in our cohort (four t(9;22) vs. 23 t(12;21) samples). To further investigate this possibility, we estimated per-class accuracy using a classical under-sampling procedure [24] in which four patients were chosen randomly within each subtype at each iteration (n = 100). This procedure increases the prediction accuracy for the t(9;22) patients to 74% thus indicating transcriptional profile specificity for this subtype (**Fig 3B**). The lower prediction accuracy for the "Other" group is concordant with the broad diversity of subtypes composing this subgroup. These results indicate that pre-B cALL subtypes have specific expression patterns that can be used to better classify tumors without resorting to classical cytogenetic methods.

We have shown that simple k-nearest neighbors classification classified cALL subtypes accurately with relatively few genes, underscoring the strength and specificity of the observed gene expression signatures. Notably, deeper examination of a few misclassified samples led to the adequate classification of the four t(12;21) that were originally missed and that were re-categorized in the proper subtype.

### Deregulated genes are enriched in bivalent chromatin marks in their promoters

**Fig 4:**
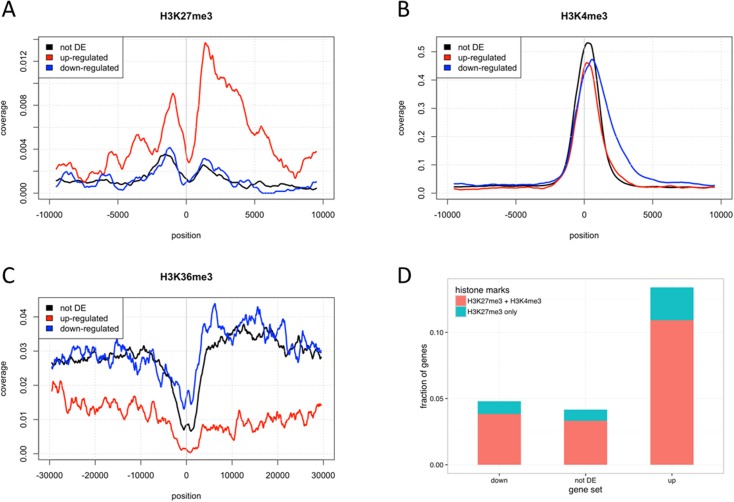
Histone mark distribution with respect to dysregulation status in pre-B cALL. (A) Relative peak coverage of H3K27me3 repressive mark. (B) Relative peak coverage of H3K4me3 activating mark. (C) Relative peak coverage of the H3K36me3 mark associated to active transcription. (D) Fraction of genes with H3K27me3 or both H3K27me3 and H3K4me3 (bivalency) near their TSS (-5kb to +5kb). Genes with an FDR<0.001 and a log2FC > 2 (or < -2) in all subtypes have been classified as up-regulated (or down-regulated). Genes not differentially expressed (not DE) include all genes with FDR>0.5. Only the most upstream TSS of each gene was considered. Histone peak data was obtained from ENCODE epigenome E031 [55].

We studied histone modification signatures associated with the DET using a publically available epigenetic ChIP-seq data for CD19^+^ primary B-cells [25]. The latter is a cell type close to our CD10^+^CD19^+^ HCB controls. When looking at genes upregulated in our dataset (see **S1 Table**), we observed an enrichment for repressive H3K27me3 marks in CD19^+^ cells near the transcription start sites (TSS) and a concordant depletion in transcription-associated H3K36me3 on these genes’ body (**Fig 4A–4C**). This indicates that these genes are not actively transcribed in normal CD19^+^ cells. Conversely, downregulated genes (see **S1 Table**) exhibit low levels of H3K27me3 and are enriched with transcription-associated H3K36me3 marks on the gene body, suggesting they are actively transcribed in CD19^+^ cells. These patterns of histone methylation further corroborate the validity of the DETs found in our analyses.

To investigate whether the observed deregulated expression in pre-B cALL is associated to promoter bivalency, we assessed the proportion of genes harboring both the H3K4me3 mark, indicating an active promoter, and the H3K27me3 mark (inactive promoter) within 5kb of their TSS. We observed a 3.28-fold enrichment (10.9% vs. 3.3%) in bivalent promoters for upregulated transcripts compared to controls (**Fig 4D**). These observations suggest that over-expression of several genes in pre-B ALL may be mediated through loss of H3K27me3 marks at bivalent domains.

**Fig 5:**
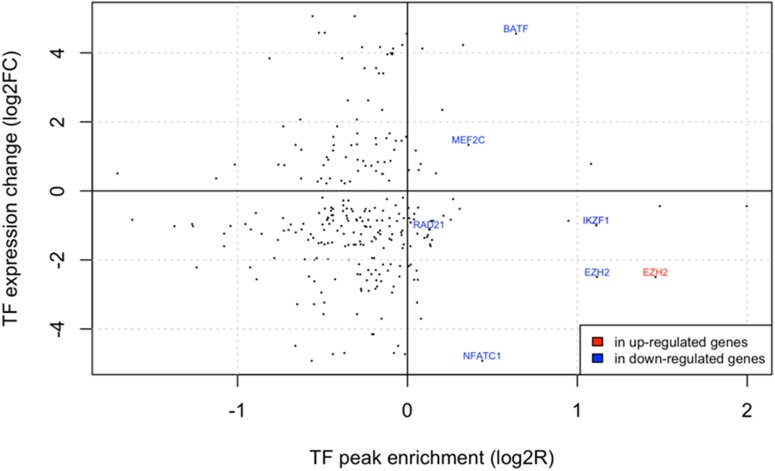
ENCODE TF peak enrichment near TSS of dysregulated genes. The y-axis corresponds to the minimal TF expression change observed among all subtypes. The x-axis corresponds to the peak enrichment ratio for genes that are up- or down-regulated in all subtypes. All TFs are represented as dots and text labels have been added when both expression change and (positive) peak enrichment are statistically significant (FDR < 0.1).

**Fig 6:**
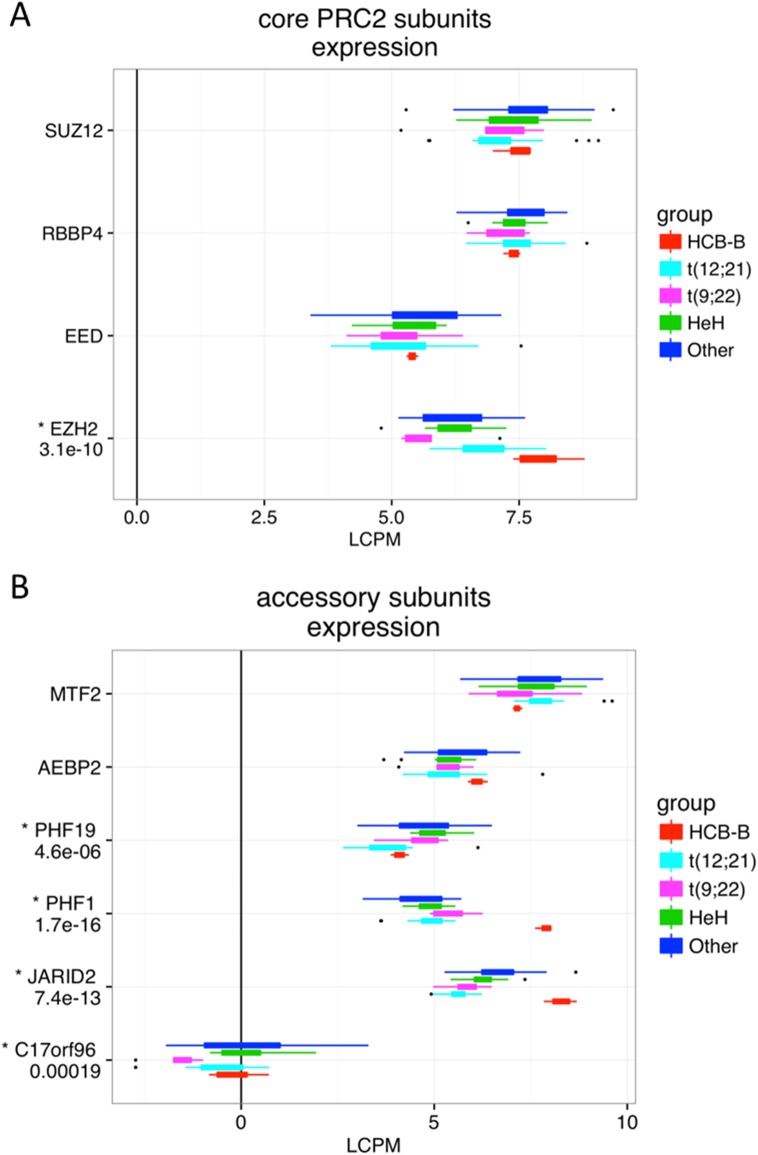
Expression distribution for core and accessory PRC2 subunits in our pre-B cALL cohort. Gene expression box plots for (A) core and (B) accessory PCR2 subunits. Thick boxes comprise observations from the first to the third quartiles in each group. Observations farther than 1.5*IQR (inter-quartile range) from these boxes boundaries are represented as dots. Genes identified as dysregulated by the edgeR analysis (FDR<1e-3) are marked with an asterisk and associated FDR values specified underneath.

We then investigated associations between global gene expression dysregulation and transcription factor (TF) binding by assessing the enrichment of 161 TFs available from ENCODE near the TSS of the deregulated genes. These ChIP-seq experiments were performed on various cell types, thus these peaks have to be considered as indicators of potential regulatory interactions. We focused on TFs whose genes are deregulated in our pre-B cALL cohort according to our analyses (**Fig 5**; **S3 Table**). We observed significant peak enrichments (FDR<0.1) for six TFs that were significantly deregulated in pre-B cALL (FDR<0.1, no logFC threshold). The strongest enrichment was observed for *EZH2*, a histone H3K27 methyltransferase part of the polycomb repressive complex 2 (PRC2) implicated in the establishment of bivalent promoters [26,27]. The transcription of *EZH2* and two other PRC2 subunits, *PHF1* and *JARID2*, was significantly downregulated in our dataset (**Fig 6**), strongly suggesting that this complex may play a key role in gene expression modulation in pre-B cALL by altering chromatin state at deregulated genes’ promoters.

**Fig 7:**
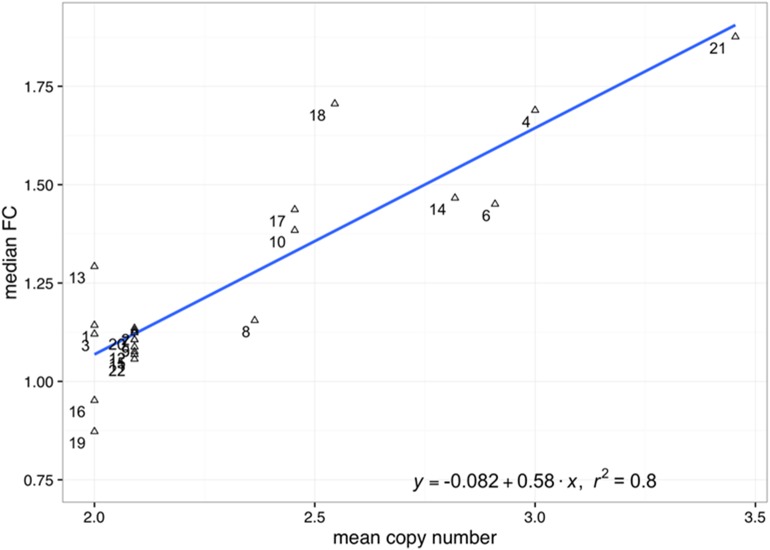
Correlation between median fold change (FC) and average copy number in the HeH group (R^2^ = 0.8). The y-axis corresponds to the chromosome median fold-change between the HeH group and HCB controls. The x-axis corresponds to the chromosome mean copy number in the HeH group. To avoid division by very small quantities, we restricted this analysis to genes expressed at >30 counts per million (CPM) in both groups. Only autosomes were included in this analysis.

We first sought for evidences of gene dosage effect in the HeH subtype by comparing the chromosome median fold change between HeH samples and HCB controls to each chromosome’s mean copy number. We observe a strong linear relationship between these two groups (Pearson’s r = 0.89; p-value<2e-8) (**Fig 7**), indicating that gene dosage indeed contributes to the transcriptional profile of HeH cALL.

**Fig 8:**
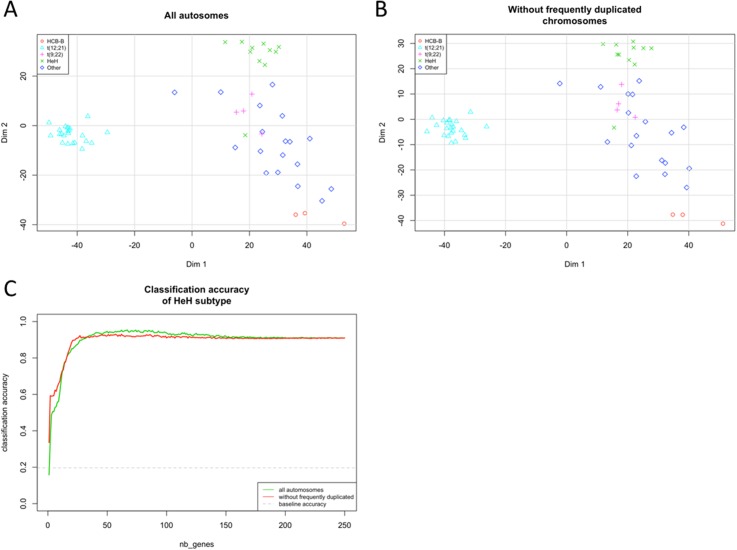
Effect of frequently gained chromosomes on inter-sample distances and KNN classification accuracy. (A) MDS plot obtained with the 500 top variance genes including all autosomes. (B) MDS plot obtained with the 500 top variance genes that are not located on chromosomes frequently gained in HeH (chr 4,6,10,14,17,18 and 21). (C) Effect on classification accuracy. The y-axis corresponds to the fraction of HeH samples correctly classified, averaged over 100 replicates. For each replicate, we sampled 50% of available genes and ordered them according to expression variance across samples. 3-nearest-neighbors classification was then performed using an incremental number of genes and Euclidean distance between samples. The baseline accuracy corresponds to random assignment of tumor subtypes within the cohort.

To quantify this contribution, we removed HeH-specific gene dosage effect by excluding all reads that mapped to chromosomes frequently gained in HeH (chromosomes 4,6,10,14,17,18, and 21) and repeated the MDS and KNN analyses. Although 143 of the 500 top variance transcripts used in the original analyses were located on excluded chromosomes and thus replaced by lower-variance ones, the MDS plots remain strikingly similar (Pearson’s r between distance matrices = 0.98) (**Fig 8A and 8B**). Concordantly, the accuracy of KNN classification for the HeH subtype using the 50 transcripts with highest variance remains similar after chromosome exclusion (92.1% before vs. 94.5% after exclusion; **Fig 8C**). Overall, these results demonstrate that gene dosage has a limited effect on the observed transcription pattern associated with the HeH subtype.

### LncRNA expression profiles classify disease subtypes

**Fig 9:**
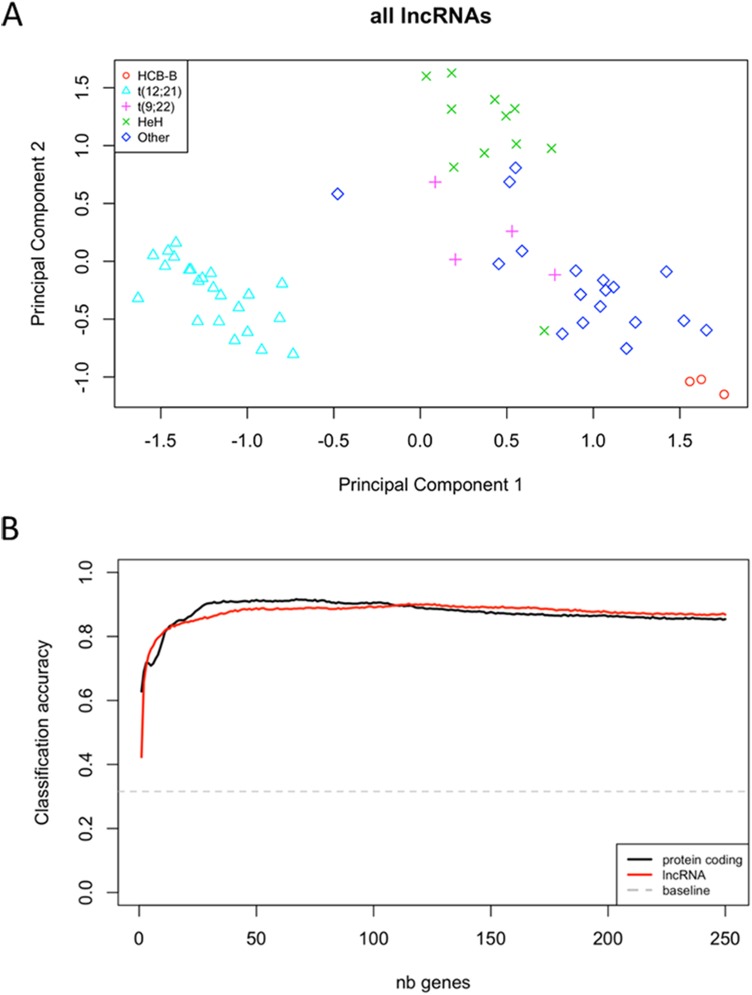
Overall accuracy of 3-nearest-neighbors classification using an increasing number of top variance genes from different biotypes. (A) Multidimensional scaling plot of distances between expression profiles only for lncRNAs. The distance between each pair of samples is the Euclidean distance between expression values (logCPM) of the 500 lncRNAs with the most variance across all samples. (B) K-nearest neighbors classification accuracy comparison between lncRNA and protein-coding transcripts. The y-axis corresponds to the fraction of samples correctly classified, averaged over 100 replicates. For each replicate, we sampled 50% of available genes and ordered them according to expression variance across samples. 3-nearest-neighbors classification was then performed using an incremental number of genes and Euclidean distance between samples. The baseline accuracy corresponds to random assignment of tumor subtypes within the cohort.

In addition to identifying hundreds of protein-coding genes differentially expressed in PreB ALL subtypes, we also characterized the expression levels of lncRNAs, which are not present on typical microarrays. By comparing the pre-B ALL subtypes with normal controls, we showed that the expression of 799 lncRNAs is specifically deregulated in pre-B cALL, 122 of which were subtype-specific. We further observed that differentially expressed lncRNAs are more likely to be subtype-specific than protein-coding genes (odds ratio = 1.72, p-value = 4.98e-6). MDS analysis and hierarchical clustering using expression levels of the lncRNAs recapitulated the subtype-specific patterns we observed with protein coding genes (**Fig 9A**). By comparing the overall KNN subtype classification accuracy of lncRNAs to that of protein-coding genes and found that they were highly similar (88.6% vs 90.4% when using the 50 highest-variance genes; **Fig 9B**), which is in agreement with a recent study [19].

Only a small proportion of lncRNAs have been functionally characterized so far and the vast majority of them have no functional annotation. Here we performed weighted gene co-expression network analysis (WGCNA) to regroup the 5000 protein-coding and lncRNA genes with the most expression variance across samples into modules based on co-expression [28,29]. This way we sought to annotate lncRNA using functional annotation enrichment within these modules

We identified 18 such modules of co-expressed genes, several of which have subtype-specific expression patterns (**S1 Fig**). Gene ontology enrichment analysis revealed significant associations (FDR<0.1) with biological processes related to cell division, T cell receptor signaling pathway, innate immune response, and nucleosomes (**S4 Table**). These data showed that lncRNA expression profiles can discriminate between pre-B cALL subtypes as well as protein-coding genes and thus could be used as subtype-specific biomarkers. Furthermore, the WGCNA analysis provided interesting leads on pre-B cALL lncRNAs’ biological functions.

## Discussion

Accurate patient stratification is the key to efficient, personalized pre-B cALL treatment. To date, childhood leukemia research has mainly focused on the expression deregulation of protein-coding genes that could be used as diagnostic and prognostics biomarkers. The human transcriptome comprises not only protein-coding mRNAs but also a large set of non-protein coding transcripts that have structural, regulatory or unknown functions [30]. In this study, we performed a whole the transcriptome analysis to discriminate pre-B cALL subtypes using both the coding and non-coding landscapes. We observed significant differences in gene expression levels between subtypes indicating that gene expression pattern could potentially be used to stratify the patient in each pre-B ALL subtype. Several such studies had previously reported that protein-coding genes’ expression profile could discriminate subtypes [7,8,21].

We showed that lncRNA gene expression patterns can classify disease subtypes as well as protein-coding genes. A recent study reported similar findings [19], but none had yet compared both approaches or leveraged the power of complete transcriptome dataset to classify pre-B cALL leukemia subtypes. Deregulation of lncRNAs has been linked to several complex human diseases, including cancer [15,31]. For instance, *MALAT1* was found to be highly expressed and associated with metastasis and poor prognosis in many cancer types [32], including non-small cell lung carcinoma [33,34] and hepatocellular carcinoma [35]. The up-regulation of several other lncRNAs, such as *HOTAIR* and *MVIH*, and the downregulation of *H19* have been associated with poor prognosis in cancers [36]. The up-regulation of lncRNA *PCA3* (*DD3*) has proven to be a reliable biomarker for prostate cancer early detection [37]. Only few lncRNAs have been directly involved in leukemogenesis. In childhood pre-B ALL, one study showed that expression of four lncRNA *BALR-1*, *BALR-2*, *BALR-6*, and *LINC0098* correlated with cytogenetic abnormalities, disease subtypes and survivals of B-ALL patients [19]. In our study, we also observed an upregulation of BALR-1 and LINC0098 in t(12;21) pre-B cALL. In addition we showed an over-expression of these two lncRNAs in the HeH subtype. *BALR-2* has been shown to be specifically upregulated in MLL-rearranged ALL [19]. Here, we showed that two patients harboring either the t(4;11) or the t(9;11) translocations were indeed associated with increased *BALR-2* expression. Of note, *BALR-2* deregulation was also observed in HeH and t(12;21) subtypes, suggesting that its overexpression may not be specific to *MLL*-rearranged leukemia subtype. *BARL-2* was identified as a modulator of the response to corticosteroid treatment, which is a cornerstone of B-ALL therapy [19]. In this regard, we failed to stratify patient according to risk (data not shown) as reported elsewhere [7]. This might be explained, at least partly by the limited number of relapsed samples in our cohort (11/56, or 19.6%), which limits the power of our clustering and classification analyses with regards to treatment outcome.

Interestingly, we found that upregulated transcripts were associated with low H3K27me3 (repressive) and high H3K36me3 (activating) marks at their promoters, indicating active transcription, while the opposite was observed for downregulated transcripts. The co-occurrence of H3K27me3 and H3K4me3 marks at promoter regions has been associated to a bivalent state allowing timely activation of developmental genes while maintaining repression in absence of differentiation signals [27]. H3K4me3 is generally considered to promote transcription, and Polycomb Repressive Complex 2 (PRC2) is responsible for repressing gene expression by depositing H3K27me3 marks on their promoters [38]. The downregulation of *EZH2* and two other PRC2 subunits, *PHF1* and *JARID2*, coupled with the epigenetic data suggests that PRC2 downregulation might be causing loss of H3K27me3-dependant expression repression and associated gene upregulation. In addition, the alteration of PRC2 function has been observed in myeloid disorders [39] and childhood ALL [40–42] thus showing specific chromatin-based gene regulation in hematological disorders.

We found that HeH samples clustered together, indicating an HeH-specific expression signature. We found a correlation between chromosomal copy number and median expression fold-change, confirming that gene dosage does indeed contribute to the observed HeH-specific transcriptional signature. The contribution of a gene dosage effect brought upon by excess chromosomes has been the subject of debate with some studies finding that gene expression changes correlate to chromosomal copy number [8,9,43,44] while others observed the opposite [9,43,44]. In this regards, we were able to classify HeH subtypes with the same accuracy once we removed the contribution of transcripts originating from duplicated chromosomes. These results indicate that the HeH subtype possess a strong transcriptional signature that is independent of gene dosage effect and involves other regulatory mechanisms.

In conclusion, we showed that pre-B cALL subtypes can be robustly and accurately discriminated using whole transcriptome data and that protein-coding and lncRNA gene expression are equivalent in doing so, in agreement with Fernando *et al*. [19], and showed evidence linking the PRC2 polycomb complex to epigenetic regulation of genes significantly deregulated in pre-B cALL.

## Material and methods

### Study subjects

Our study cohort consisted of 56 pre-B cALL patients (28 females and 28 males) with a mean age at diagnosis of 6.1±3.6 years. All subjects were French-Canadians of European descent diagnosed in the Division of Hematology-Oncology at the Sainte-Justine Hospital (Montreal, Canada) and part of the Quebec childhood ALL cohort (QcALL) [45]. Cohort details are provided in **Table 1**. CD10^+^CD19^+^ cells isolated from human cord blood were used as controls. Briefly, after being isolated using a Ficoll-Paque gradient fragmentation, PBMCs were positively selected using MACS Separation with CD19 MicroBeads (Miltenyi Biotec). Cell sorting was performed on the CD19+ cells using CD19-PE and CD10-FITC antibodies (Miltenyi Biotec). Purity was >90%. The Sainte-Justine Institutional Review Board approved the research protocol, and written informed consent was obtained from all participating individuals and/or their parents.

### Sample preparation and RNA sequencing analyses

Total RNA was extracted from white blood cell pellets obtained from bone marrow and peripheral blood at diagnosis using the mirVana Isolation kit (Ambion) according to manufacturer’s protocol. Following a DNAse treatment to remove possible contamination by genomic DNA, ribosomal RNAs were removed using the RiboMinus Eukaryote kit (Invitrogen). cDNA libraries were prepared using the SOLiD Total RNA-seq kit based on manufacturer’s protocol and sequenced on the Life Technologies SOLiD 4/5500 System (paired-end: 50x35bp and 75x35bp). Reads were aligned to the human genome (hg19) using the Lifescope Genomic Analysis Software (Applied Biosystems; Whole Transcriptome Analysis pipeline, default parameters). Expression levels by gene were determined with the HTseq-count software [46] using gene models from Ensembl75 combined with (non-overlapping) lncRNA transcripts provided in Casero et al. [47]. The identification of differentially expressed transcripts (DET) relative to HCB controls was done using the Generalized Linear Model implemented in the edgeR package [48].

Genes were considered subtype-specific when their average expression was at least 4-fold higher or lower in a given subtype relative to the average expression in all other subtypes. The subtype specificity score of a gene was defined as the difference between its average expression in a specific subtype and its average expression in the closest subtype.

The analysis of the public expression microarray data [20] was performed with the Limma package [48] and eBayes function. The datasets were downloaded from the Gene Expression Omnibus website (GEO Series number GSE56599).

Whole transcriptome datasets are available on the Gene Expression Omnibus (GEO) under accession number GSE89071.

### Statistical analyses

We used the false discovery rate (FDR) to correct for multiple hypothesis testing [49]. P-values were obtained using standard likelihood ratio tests and corrected using FDR. Coefficients have been included in the model to alleviate potential batch effects. A corrected P-value ≤ 0.001 was used as threshold for selecting significantly differentially expressed genes. We considered considering an incremental number of genes selected among those with highest expression variance across all samples. This procedure was repeated 100 times using a random subsample (50%) from all available transcripts at each iteration to get robust estimates.

### Multidimensional scaling (MDS) and K-nearest-neighbors analyses

Log Count per Million Mapped Reads (LCPM) were obtained using edgeR's cpm function [50] and normalised using the upper-quartile method. We performed MDS analyses using the 500 top variance genes using edgeR’s plotMDS function with a prior count of 1.

K-nearest-neighbors classification was performed using the knn function [23] from the Class package (https://cran.r-project.org/). To reduce variance of accuracy estimates, the classification procedure was repeated 100 times using a random subsample (50%) from all available genes at each iteration. For the balanced class predictions, four samples from each class were chosen randomly at each iteration. We used the “gene_biotype” attribute from Ensembl75 annotations to define the sets of lncRNAs and protein coding genes.

### Gene dosage effect in the HeH subtype

LCPM were obtained as above using read counts from either all autosomes or restricted to autosomes that are not frequently gained in the HeH subtype (1, 2, 3, 5, 7, 8, 9, 11, 12, 13, 15, 16, 19, 20, and 22). For each chromosome, the fold-change between average expression in the HeH subtype and the HCB control group for all genes was computed. The median values were plotted against the mean chromosome copy number in the HeH subtype and a linear regression was performed using the lm function in R. The MDS and KNN analyses were performed as previously described.

### Histone marks and TF peak enrichment

Histone marks for CD19^+^ primary B-cell were obtained from ENCODE epigenome E031 [25]. The binding sites for 161 ENCODE transcription factors [51] were obtained from the file "wgEncodeRegTfbsClusteredWithCellsV3" downloaded from the UCSC website (https://genome.ucsc.edu/). Histone mark or TF binding enrichments near deregulated genes’ promoter regions was determined using the GenomicRange package [52] and the phyper function.

### Weighted gene co-expression network analysis (WGCNA) analysis

Genes were ranked according to expression variance across samples. Topological overlap and dissimilarity matrices for the top 5000 genes were determined using WGCNA R package [29]. Hierarchical clustering was then performed using the ward.D2 function [53] and modules determined using WCGNA’s default dissimilarity threshold (0.25). Gene ontology enrichment within each module was performed using the topGO package [54].

### RT-PCR validation of t(12;21)[*ETV6*/*RUNX1*] translocation

Total RNA was extracted (as above) from the patients 5, 73, and 220’s bone marrow at diagnosis. RNA was reverse transcribed into cDNA using the QuantiTect® Reverse Transcription Kit (Qiagen). PCR were performed using KOD Polymerase as described above. Amplified fragments were analyzed on the Agilent 2100 Bioanalyzer Instrument and by Sanger sequencing (McGill University and Genome Quebec Innovation Centre).

This study was performed in 2016.

## Supporting information

S1 Fig Weighted gene co-expression network analysis (WCGNA) module-subtype relationship. The blue-red scale indicates the correlation between each module's eigengene’s expression and sample's membership for a specific tumor subtype (0 or 1). Correlation and corrected p-value (FDR) are indicated in a cell when the FDR is below 0.1.
(TIF)

S1 Table Gene relative expression data for ALL disease subtypes vs. CD19+CD10+ controls. This table contains gene expression data (logCPM), log2 fold-change by disease subtype (vs. HCB controls) and expression subtype-specificity score. See Methods for details.
(XLSX)

S2 Table Differentially expressed genes common to both Lee et al.'s and our datasets. This table contains log2 fold-changes for genes differentially expressed common to both our dataset and Lee et al.’s. See Methods for details.
(XLSX)

S3 Table Transcription factor binding site enrichment on differentially expressed transcripts' promoters for transcription factors specifically deregulated in pre-B cALL. This table shows transcription factor binding site enrichment (from ENCODE data) in the promoters of transcription factors differentially expressed in pre-B cALL samples. See Methods for details.
(XLSX)

S4 Table Gene ontology term enrichment for WCGNA clusters. This table shows gene ontology (GO) term enrichment for WCGNA modules. See Methods for details.
(XLSX)
