# Supplementary figures and images for: Specific expression of novel long non-coding RNAs in high-hyperdiploid childhood acute lymphoblastic leukemia
Source: PLoS One. 2017 Mar 27;12(3):e0174124. doi: 10.1371/journal.pone.0174124 (PMC5367703; doi:10.1371/journal.pone.0174124)

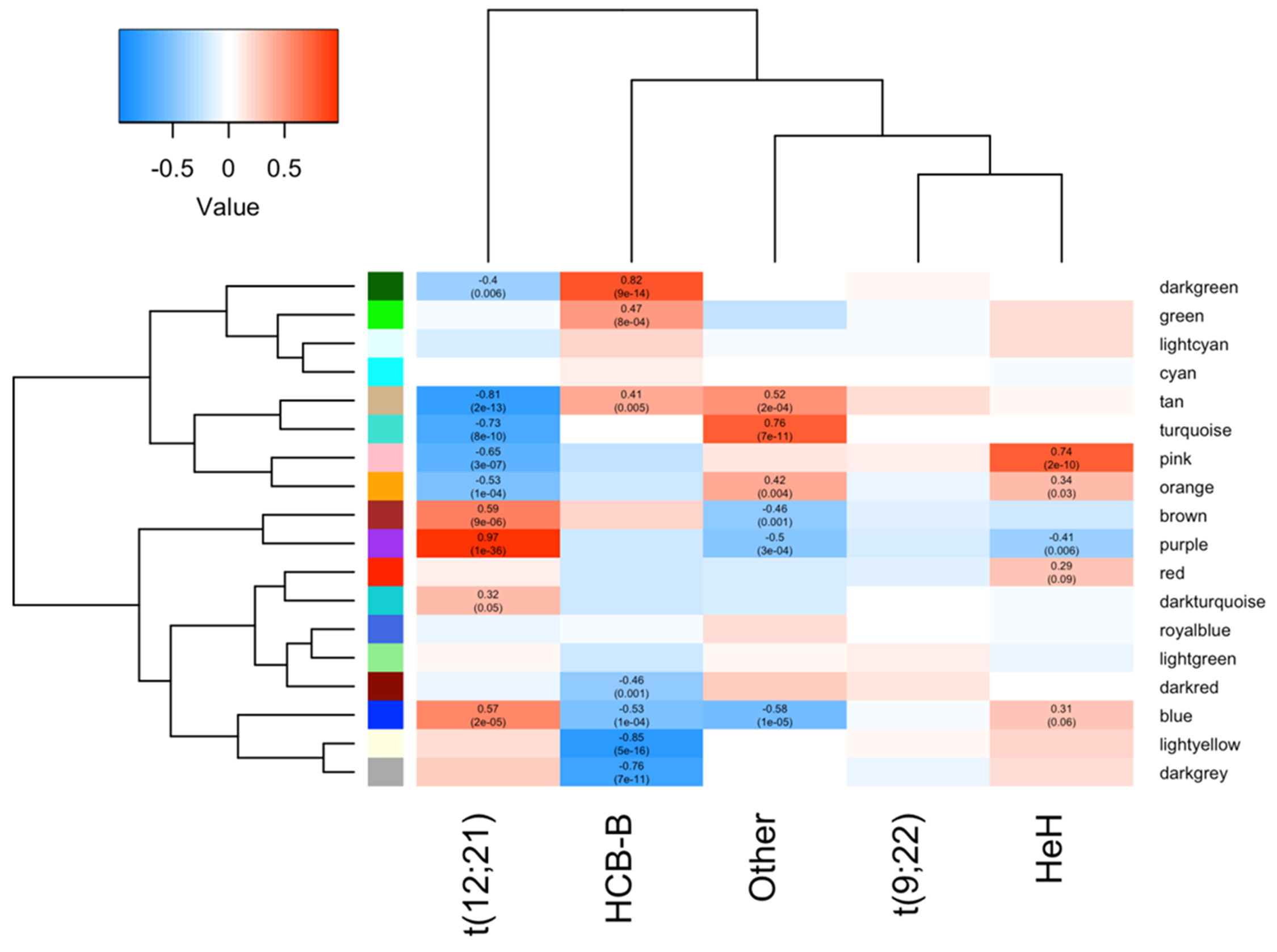

Supplement: S1 Fig — The blue-red scale indicates the correlation between each module's eigengene’s expression and sample's membership for a specific tumor subtype (0 or 1). Correlation and corrected p-value (FDR) are indicated in a cell when the FDR is below 0.1. (TIF) [file pone.0174124.s001.tif]
